# Beyond Monogenic Inheritance: Emerging Genetic Architecture of Amyotrophic Lateral Sclerosis

**DOI:** 10.3390/ijms27209350

**Published:** 2026-10-21

**Authors:** Sherin Ramadan, Emanuele Panza, Antonio Orlacchio

**Affiliations:** 1 Department of Medicine and Surgery, University of Perugia, 06132 Perugia, Italy; 2 Department of Medical and Surgical Sciences, University of Bologna, 40138 Bologna, Italy; 3 Medical Genetics Unit, IRCCS Bologna University Hospital, 40138 Bologna, Italy; 4 Perugia University Hospital, 06132 Perugia, Italy

**Keywords:** amyotrophic lateral sclerosis, genetic architecture, whole-genome sequencing, non-coding variants, oligogenic inheritance, precision medicine

## Abstract

Amyotrophic lateral sclerosis (ALS) is a fatal neurodegenerative disorder characterized by marked genetic and phenotypic heterogeneity. Although pathogenic variants in major ALS genes, including *C9ORF72*, *SOD1*, *TARDBP*, and *FUS*, have provided fundamental insights into disease pathogenesis, many familial and most sporadic cases remain without a molecular diagnosis, suggesting a genetic architecture extending beyond coding variants and monogenic inheritance. Advances in genomic technologies have revealed contributions from deep-intronic and regulatory variants, structural rearrangements, repeat expansions, and other forms of genomic variation incompletely captured by standard diagnostic approaches. Evidence also indicates that combinations of rare variants with moderate or incomplete penetrance may contribute to disease susceptibility and clinical variability, supporting a multilayer model of ALS genetics. Population-specific variation and convergence of genetically diverse defects on common molecular pathways further complicate variant interpretation and disease classification. This review examines emerging components of ALS genetic architecture, including non-coding variation, structural variants, repeat expansions, oligogenic burden, ancestry-related gaps, and convergent neurodegenerative pathways. Integrating these genomic layers with functional and transcriptomic evidence may improve variant interpretation, diagnostic yield, patient stratification, and the development of mechanism-based precision therapies.

## 1. Introduction

Amyotrophic lateral sclerosis (ALS) is a progressive and fatal neurodegenerative disorder characterized by degeneration of upper and lower motor neurons, leading to muscle weakness, paralysis, and ultimately respiratory failure. Increasing evidence indicates that ALS represents a clinically and biologically heterogeneous syndrome rather than a single disease entity, with substantial variability in age at onset, site of onset, cognitive involvement, progression rate, and survival reflecting the interplay of multiple genetic and non-genetic factors [1,2,3].

Traditionally, ALS has been classified as familial (fALS) or sporadic (sALS). However, this distinction has become increasingly blurred following the identification of pathogenic variants in *C9ORF72*, *SOD1*, *TARDBP*, and *FUS* in both familial and apparently sporadic patients. Reduced penetrance, *de novo* variants, incomplete pedigrees, and genetic modifiers frequently obscure Mendelian inheritance, supporting the view that familial and sporadic ALS represent different positions along a continuous spectrum of genetic susceptibility rather than distinct biological entities [4,5,6,7].

The discovery of *SOD1* in 1993 initiated the molecular era of ALS genetics, while the identification of the intronic GGGGCC repeat expansion in *C9ORF72* established a direct genetic link between ALS and frontotemporal dementia and demonstrated that highly pathogenic mutations may reside outside protein-coding regions [8,9,10]. Subsequent advances in genome-wide association studies and next-generation sequencing expanded the number of ALS-associated genes and identified convergent biological pathways involving RNA metabolism, proteostasis, autophagy, mitochondrial function, nucleocytoplasmic transport, axonal trafficking, and neuroinflammation [1,6,11,12].

Despite these advances, currently recognized disease genes explain only part of ALS heritability. Increasing evidence indicates that non-coding variants, structural rearrangements, repeat expansions, regulatory variation, oligogenic inheritance, common susceptibility alleles, and ancestry-specific genetic factors contribute to disease risk but remain incompletely detected by conventional diagnostic approaches [4,6,7,12].

This review examines the emerging multilayer genetic architecture of ALS beyond the traditional monogenic paradigm. We discuss hidden genomic variation, cumulative genetic risk, and convergent pathogenic pathways, highlighting how integration of genomic, transcriptomic, and functional data is reshaping disease classification, improving molecular diagnosis, and supporting the development of precision medicine.

The primary focus of this review is the emerging genetic architecture of ALS. Cellular and molecular mechanisms are discussed insofar as they provide a functional framework for understanding how heterogeneous forms of genetic variation converge on a limited number of disease-relevant biological processes. Thus, rather than providing a comprehensive review of ALS cell biology, we aim to connect different layers of genetic variation with their principal downstream functional consequences and potential translational implications.

## 2. Current Genetic Landscape of ALS

The identification of ALS-associated genes has transformed the understanding of disease pathogenesis and established genetics as a cornerstone of molecular diagnosis, patient stratification, genetic counseling, and therapeutic development. Since the discovery of *SOD1* in 1993, linkage studies, genome-wide association studies (GWAS), next-generation sequencing (NGS), and international sequencing consortia have expanded the ALS genetic landscape to more than 40 associated genes. However, only a subset currently fulfills robust criteria for disease causality, whereas others are better classified as susceptibility genes, genetic modifiers, or candidate loci requiring further validation [6,7,8,13,14,15,16,17,18].

Pathogenic variants in *C9ORF72*, *SOD1*, *TARDBP*, and *FUS* account for most genetically defined familial ALS and a smaller proportion of apparently sporadic cases. Together with subsequent discoveries involving *TBK1*, *OPTN*, *SQSTM1*, *VCP*, *NEK1*, *KIF5A*, *SPG11*, *CHCHD10*, and *ANXA11*, these genes have identified the principal biological processes underlying ALS, including RNA metabolism, proteostasis, autophagy, mitochondrial homeostasis, axonal transport, nucleocytoplasmic trafficking, and neuroinflammatory signaling [1,3,6,11,19,20,21,22,23,24,25,26,27,28,29,30]. The major ALS-associated genes and their biological functions are summarized in Table 1.

Genetic-evidence categories follow the current ALSoD framework and indicate the strength of evidence linking variants in each gene to ALS; classifications may evolve as additional genetic and functional evidence becomes available [17].

Despite these advances, defining pathogenicity remains challenging. The clinical significance of many variants depends on segregation data, population frequency, functional studies, and independent replication rather than sequence information alone. This is particularly relevant for genes such as *NEK1*, *TBK1*, and several recently proposed ALS genes, in which variant-specific pathogenicity is often difficult to establish [12,18,28].

Genotype–phenotype correlations are similarly complex. Variants in the same gene may produce substantial differences in age at onset, progression, cognitive involvement, and survival, reflecting the influence of mutation-specific effects, genetic modifiers, and additional biological factors. Consequently, identical primary mutations do not necessarily result in comparable clinical phenotypes. Moreover, *KIF5A* mutations may present phenotypes ranging from hereditary spastic paraplegia to ALS-like syndromes with upper and lower motor neuron involvement, further illustrating the clinical continuum of motor neuron diseases [3,6,35,36].

**Figure 1:**
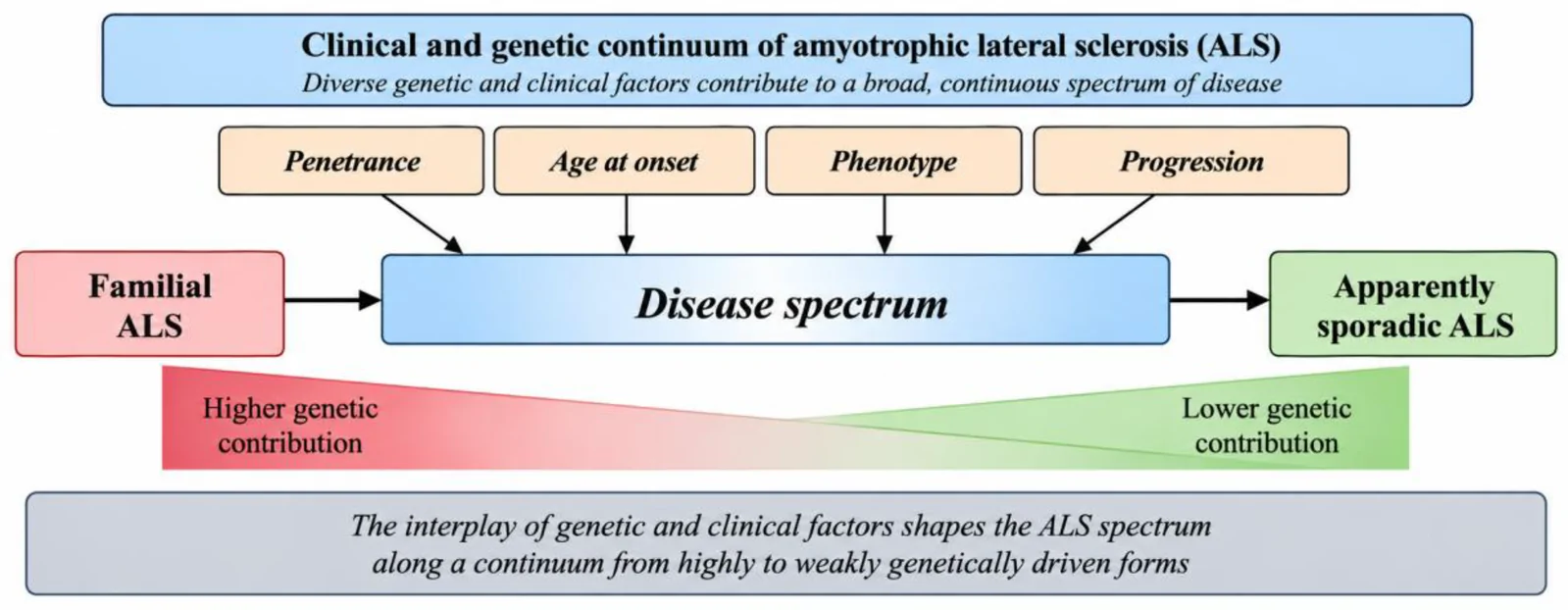
Clinical and genetic continuum of ALS. Familial and apparently sporadic ALS represent a continuous disease spectrum shaped by genetic contribution, penetrance, age at onset, phenotype, and progression.

The widespread adoption of whole-genome sequencing, long-read sequencing, and transcriptomic analyses is expanding the spectrum of detectable genetic variation beyond conventional coding mutations. Nevertheless, many familial and most sporadic ALS cases remain genetically unexplained, indicating that the current catalogue of disease genes represents only part of the underlying genetic architecture. This observation provides the rationale for examining hidden genomic variation and multilayer genetic risk in the following sections (Figure 1).

## 3. Beyond Coding Variants

**Figure 2:**
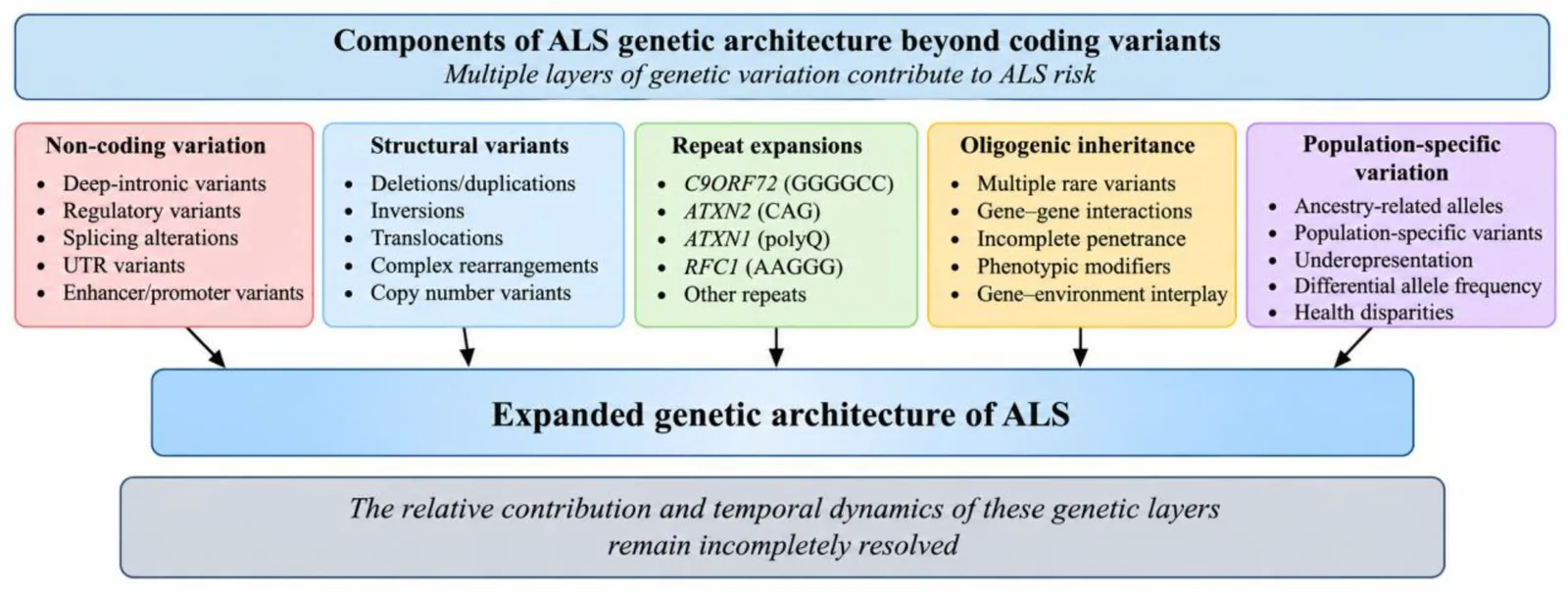
Components of ALS genetic architecture beyond coding variants. Non-coding variants, structural variants, repeat expansions, oligogenic inheritance, and population-specific variation contribute to an expanded genetic architecture whose relative contributions remain incompletely resolved.

Despite major advances in ALS gene discovery, a substantial proportion of patients remain without a molecular diagnosis after conventional genetic testing. Current diagnostic strategies rely mainly on targeted gene panels or whole-exome sequencing (WES), which interrogate only the protein-coding portion of the genome, representing approximately 1–2% of human DNA. Increasing evidence indicates that non-coding variants, structural rearrangements, repeat expansions, and other forms of hidden genomic variation contribute to the unresolved heritability of ALS and frequently escape standard diagnostic pipelines [6,37,38,39]. The principal classes of hidden genomic variation are summarized in Figure 2.

### 3.1. Non-Coding Variants

The non-coding genome contains regulatory elements that control transcription, RNA processing, transcript stability, chromatin organization, and translation. Variants affecting promoters, enhancers, untranslated regions, introns, or non-coding RNAs may therefore alter gene function without changing protein sequence and are generally missed by routine exome-based analyses [6,37].

Two distinct mechanisms must be distinguished when considering abnormal splicing in ALS. Inherited non-coding variants may directly disrupt splice sites or cis-regulatory elements, alter splicing enhancers or silencers, or promote intron retention and cryptic exon inclusion. By contrast, aberrant splicing may also arise secondarily from dysfunction of RNA-binding proteins, without a pathogenic DNA variant at the affected transcript locus. Both mechanisms can lead to transcript degradation or abnormal protein products despite an intact coding sequence, but they have different implications for genetic causality and variant interpretation. Transcript-level analyses are therefore essential to establish the functional consequences of candidate non-coding variants.

TDP-43 provides a prominent example of the second mechanism. Its nuclear depletion and cytoplasmic aggregation characterize most ALS cases, and loss of nuclear TDP-43 activity causes widespread cryptic exon inclusion, aberrant splicing, and altered RNA processing [40,41,42,43,44,45]. These transcriptomic changes are downstream consequences of TDP-43 protein dysfunction and should not, by themselves, be classified as inherited non-coding mutations.

Among its best-characterized downstream targets is *STMN2*, a key regulator of axonal maintenance and regeneration. TDP-43 depletion induces cryptic splicing and premature polyadenylation of *STMN2*, reducing full-length transcript expression, whereas restoration of normal splicing rescues axonal defects in experimental models [46,47,48]. Thus, *STMN2* mis-splicing primarily illustrates a consequence of nuclear TDP-43 loss rather than an established effect of an inherited *STMN2* risk variant.

*UNC13A* illustrates a distinct interaction between inherited susceptibility and acquired RNA-processing dysfunction. Common intronic ALS risk variants in *UNC13A* enhance cryptic exon inclusion when nuclear TDP-43 function is lost, leading to nonsense-mediated decay and reduced UNC13A protein expression [49,50]. The variants do not simply cause constitutive aberrant splicing independently of TDP-43 pathology; instead, they modify the extent of a TDP-43-dependent splicing defect. This provides a mechanistic example of how germline non-coding variation can interact with a disease-associated cellular state.

More broadly, transcriptomic studies have identified widespread cryptic exons, intron retention, alternative polyadenylation, and abnormal transcripts associated with TDP-43 dysfunction, some generating cryptic peptides detectable in nervous tissue and cerebrospinal fluid, highlighting their potential diagnostic and biomarker value [45,51].

Taken together, these findings distinguish inherited non-coding variants that directly alter RNA regulation from secondary transcriptomic abnormalities caused by TDP-43 dysfunction. *STMN2* exemplifies a downstream TDP-43-dependent splicing defect, whereas *UNC13A* demonstrates how germline intronic risk alleles can potentiate that defect. This distinction is important when interpreting non-coding variants and assigning genetic causality in ALS.

### 3.2. Structural Variants

Structural variants (SVs), including copy-number variants, insertions, deletions, inversions, duplications, translocations, mobile-element insertions, and complex genomic rearrangements, represent another underrecognized source of ALS genetic variation.

SVs may disrupt coding sequences, alter gene dosage, modify chromatin architecture, reposition regulatory elements, or affect long-range transcriptional regulation. Although early studies suggested that common copy-number variants contribute only modestly to ALS risk, whole-genome sequencing has revealed rare structural alterations affecting both established and candidate ALS genes [52,53,54].

Interpretation remains challenging because many SVs are individually rare, population-specific, and enriched within repetitive genomic regions. Demonstrating pathogenicity requires integration of segregation data, population frequency, transcriptomic consequences, and functional validation. Their characterization is further complicated by ancestry-specific differences in genomic architecture, which remain poorly represented in existing reference datasets [55].

Short-read sequencing has limited sensitivity for many classes of structural variation, whereas long-read sequencing, optical genome mapping, and improved bioinformatic algorithms substantially improve breakpoint resolution and variant characterization. These complementary technologies are expected to increase diagnostic yield and clarify the contribution of structural variation to ALS susceptibility [37,39,54].

### 3.3. Repeat Expansions

Repeat expansions represent one of the clearest examples of pathogenic variation beyond coding regions. The discovery of the intronic GGGGCC repeat expansion in *C9ORF72* fundamentally changed ALS genetics by demonstrating that highly penetrant disease-causing mutations may occur within non-coding DNA [8,9,10].

The *C9ORF72* expansion contributes through multiple mechanisms, including haploinsufficiency, toxic RNA foci, sequestration of RNA-binding proteins, and repeat-associated non-AUG translation producing neurotoxic dipeptide-repeat proteins. Unlike conventional sequence variants, repeat expansions remain difficult to characterize using short-read sequencing because repeat length, sequence composition, interruptions, methylation status, and somatic instability cannot be accurately resolved.

Long-read sequencing now enables direct characterization of expanded alleles, providing information on repeat size, sequence purity, methylation, and haplotype structure, all of which may influence penetrance and phenotype [56,57,58]. Somatic instability further increases complexity, as repeat length may differ substantially among blood, spinal cord, and distinct brain regions.

Although *C9ORF72* remains the best-established ALS repeat expansion, intermediate-length *ATXN2* CAG expansions consistently increase ALS susceptibility and interact biologically with TDP-43 dysfunction [59]. Intermediate *C9ORF72* alleles and expansions in other genes, including *NIPA1*, have also been proposed as susceptibility factors or modifiers, although their pathogenic significance remains less well established [60,61,62,63,64].

Overall, non-coding variants, structural rearrangements, and repeat expansions represent complementary mechanisms contributing to the missing heritability of ALS. Their principal characteristics, detection strategies, and clinical implications are summarized in Table 2.

Continued integration of whole-genome sequencing, long-read sequencing, transcriptomics, and functional genomics will be essential to achieve a more complete understanding of ALS genetic architecture.

## 4. Oligogenic Burden and Cumulative Genetic Risk

The traditional view of ALS as a predominantly monogenic disorder has progressively evolved toward a more complex genetic model in which disease susceptibility often reflects the cumulative contribution of multiple genetic factors. Although highly penetrant mutations remain the primary cause of many familial cases, increasing evidence indicates that combinations of rare pathogenic variants, intermediate-risk alleles, common susceptibility loci, and regulatory variants jointly influence disease risk, penetrance, age at onset, and clinical heterogeneity [6,7,12].

### 4.1. Oligogenic Inheritance

Oligogenic inheritance describes a model in which two or more variants in different genes interact to influence expression of disease. Unlike classical Mendelian inheritance, no single variant is necessarily sufficient to determine phenotype; instead, the cumulative burden of multiple genetic alterations contributes to disease development. This framework provides a plausible explanation for incomplete penetrance, marked intrafamilial variability, and apparently sporadic cases carrying variants in established ALS genes.

Multiple sequencing studies have identified ALS patients harboring pathogenic or likely pathogenic variants in more than one disease-associated gene, including combinations involving *C9ORF72*, *SOD1*, *TARDBP*, *FUS*, *TBK1*, *OPTN*, *SQSTM1*, and *NEK1*. Although individual combinations are rare, their recurrent observation across independent cohorts supports the existence of cumulative genetic effects rather than isolated coincidental findings [6,65,66,67,68].

Interpreting oligogenicity remains challenging because the pathogenic contribution of each variant cannot be assessed independently. Functional interactions between genes, incomplete penetrance, ancestry-specific allele frequencies, and limited statistical power complicate the distinction between true biological interactions and random co-occurrence. Consequently, robust evidence increasingly requires integrated genetic, functional, and segregation analyses rather than simple variant counting.

### 4.2. Polygenic Risk

In addition to rare variants, common genetic variation contributes to ALS susceptibility. Genome-wide association studies have identified numerous loci that individually confer only modest effects but collectively influence disease risk. Polygenic risk scores (PRS) aggregate the effects of these variants into a quantitative estimate of inherited susceptibility and help explain part of the genetic architecture not accounted for by rare mutations alone [12,69,70].

Current ALS PRS captures only part of the polygenic contribution to disease susceptibility and currently provides insufficient predictive performance for routine individual-level clinical risk prediction [12,71]. Earlier genome-wide association studies also identified the chromosome 9p21 locus as a susceptibility region for sporadic ALS [72]. Nevertheless, PRS provides insight into disease biology and may contribute to genetic stratification when integrated with rare pathogenic variants, family history, and other relevant factors. Rather than representing competing models, rare high-impact mutations and polygenic susceptibility appear to operate along the same biological continuum.

Importantly, PRS performance and transferability vary across ancestral populations because most underlying ALS GWAS have been conducted predominantly in individuals of European ancestry. Ancestry-related disparities extend beyond PRS performance and affect gene and variant discovery, allele-frequency estimation, and variant interpretation. The relative contribution of major ALS genes and individual pathogenic variants can differ across populations, while underrepresentation in population reference datasets may increase uncertainty in rare-variant classification. Larger ancestrally diverse cohorts, ancestry-appropriate reference datasets, and multi-ancestry analytical approaches are therefore required to improve the generalizability and equity of genetic diagnosis and risk assessment [55,71].

### 4.3. Integrating Rare and Common Genetic Variation

Emerging evidence supports a multilayer model of ALS genetics in which highly penetrant mutations, rare variants of moderate effect, common susceptibility alleles, non-coding variants, structural rearrangements, repeat expansions, and environmental factors collectively shape disease susceptibility and phenotypic expression. Rather than acting independently, these genetic components contribute to a cumulative risk that influences penetrance, age at onset, disease progression, cognitive involvement, and survival. This conceptual framework bridges the traditional distinction between familial and sporadic ALS, supporting the view that both represent different positions along a continuous spectrum of genetic susceptibility rather than biologically distinct entities [6,7,12].

This integrated model also explains several longstanding observations in ALS, including incomplete penetrance, marked intrafamilial variability, apparently sporadic cases carrying pathogenic variants, and substantial phenotypic heterogeneity among individuals harboring the same pathogenic variant.

Environmental exposures may further contribute to the multilayer architecture of ALS. Epidemiological studies have investigated occupational, lifestyle, and environmental exposures as potential modifiers of ALS susceptibility and progression, leading to models in which genetic susceptibility, ageing, and cumulative environmental exposures interact over the life course. However, evidence for specific gene–environment interactions in ALS remains limited and sometimes inconsistent. Robust demonstration is complicated by heterogeneous and often retrospectively assessed exposures, long latency between exposure and disease onset, and the limited availability of large cohorts combining detailed genomic and longitudinal exposure data. Accordingly, environmental factors should currently be regarded as potential components of a multifactorial susceptibility model rather than established deterministic modifiers of specific ALS genotypes [1,3].

Future studies integrating whole-genome sequencing, long-read sequencing, transcriptomics, epigenomics, functional genomics, polygenic risk analyses, and well-characterized environmental exposure data will be essential to define the relative contribution and interaction of these components and improve variant interpretation and disease-risk modeling. Such multidimensional approaches may increase diagnostic yield and refine molecular classification, but their clinical utility requires prospective validation.

## 5. Convergent Pathogenic Pathways

The remarkable genetic heterogeneity of ALS contrasts with the relatively limited number of biological processes disrupted across patients. Although more than 40 ALS-associated genes have been identified, many converge on shared cellular pathways, indicating that genetically distinct defects ultimately promote motor neuron degeneration through common molecular mechanisms rather than isolated gene-specific events [1,3,6,11,73].

**Figure 3:**
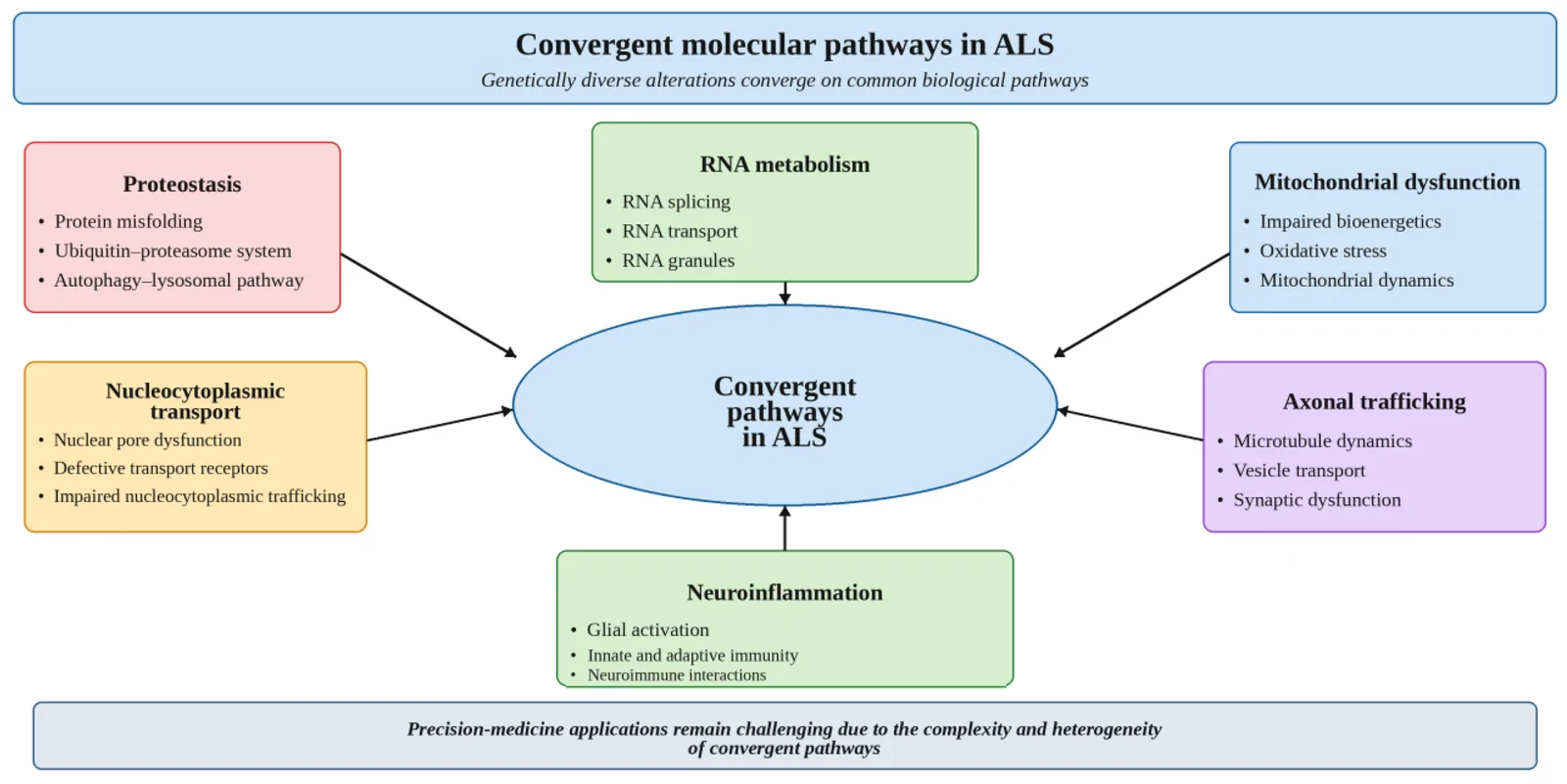
Convergent molecular pathways in ALS. Genetically diverse alterations converge on interconnected processes involving RNA metabolism, proteostasis, nucleocytoplasmic transport, mitochondrial dysfunction, axonal trafficking, and neuroinflammation; their relative contribution may vary across genetic backgrounds and disease stages.

This convergence provides a unifying framework for ALS pathogenesis. Individual proteins frequently participate in multiple biological processes, while dysfunction of one pathway often propagates to others. Consequently, abnormalities in RNA metabolism, proteostasis, autophagy, mitochondrial homeostasis, axonal transport, and neuroinflammatory signaling should be viewed as components of an integrated pathogenic network rather than independent mechanisms (Figure 3).

### 5.1. RNA Metabolism and Proteostasis

Disruption of RNA metabolism represents one of the defining molecular features of ALS. Several ALS-associated RNA-binding proteins—including TDP-43 (encoded by *TARDBP*), FUS, HNRNPA1, HNRNPA2B1, MATR3, and TIA1—regulate transcription, pre-mRNA splicing, RNA transport, stress-granule dynamics, local translation, and transcript stability. Their dysfunction can therefore affect broad transcriptomic programs rather than a single downstream target [11,42,43,74,75].

Nuclear depletion and cytoplasmic aggregation of TDP-43 characterize most ALS cases but are not universal: *SOD1*- and *FUS*-associated ALS are important neuropathological exceptions. Loss of its nuclear function disrupts RNA processing through cryptic exon inclusion, aberrant splicing, altered polyadenylation, transcript destabilization, and defective RNA transport, whereas cytoplasmic aggregates contribute to proteotoxic stress [44,46,47].

The major genetic mechanisms are therefore convergent but not interchangeable. *SOD1*-associated ALS is primarily linked to mutant SOD1 misfolding and proteotoxicity, whereas FUS-associated ALS involves abnormal localization and aggregation of the FUS RNA-binding protein, with FUS rather than TDP-43 pathology in characteristic cases [8,16]. *C9ORF72* repeat expansions imply several potentially interacting mechanisms, including repeat-containing RNA toxicity, dipeptide-repeat protein production, and reduced *C9ORF72* function [9,10]. By contrast, variants in *TBK1*, *OPTN*, and *SQSTM1* implicate selective autophagy, ubiquitin-dependent cargo recognition, and mitochondrial quality control, although the relative contribution of these processes differs across models and variants [20,23,27]. Thus, shared downstream disturbances in RNA homeostasis, protein quality control, and organelle clearance do not imply a single initiating lesion or uniform TDP-43 pathology across all ALS genotypes.

Among the most relevant downstream targets are *STMN2* and *UNC13A*. Loss of nuclear TDP-43 induces premature polyadenylation and reduced expression of *STMN2*, a protein required for axonal maintenance and regeneration, whereas correction of its mis-splicing restores axonal growth in experimental models [46,47,48]. Likewise, TDP-43 depletion promotes cryptic exon inclusion within *UNC13A*, resulting in nonsense-mediated decay. Common ALS-associated variants enhance this process, providing a direct mechanistic link between inherited susceptibility and acquired TDP-43 pathology [49,50].

RNA dysregulation also alters stress-granule dynamics. Under physiological conditions, stress granules transiently sequester untranslated mRNAs during cellular stress, and several ALS-associated RNA-binding proteins contain low-complexity domains that facilitate phase separation. Disease-associated perturbations can alter condensate dynamics and, under specific experimental conditions, favor persistent assemblies. However, whether stress granules are a necessary precursor of pathological TDP-43 aggregation across ALS remains unresolved. These observations support a context-dependent relationship between RNA dysregulation, phase separation, and proteostasis rather than a uniform linear sequence of events [11,75].

### 5.2. Protein Degradation and Autophagy

Maintenance of protein homeostasis depends on the coordinated activity of the ubiquitin–proteasome system and the autophagy–lysosome pathway. ALS-associated genes, including *TBK1*, *SQSTM1*, *OPTN*, *VCP*, and *UBQLN2*, encode proteins involved in selective autophagy, ubiquitin-mediated protein degradation, lysosomal trafficking, or protein quality control. Primary genetic studies implicate *OPTN*, *SQSTM1*, and *TBK1* in ALS [20,23,27], whereas experimental work demonstrates distinct roles for proteasomal and autophagic degradation in TDP-43 clearance [76]. These findings link impaired protein and organelle quality control to ALS-associated proteotoxic stress.

Early biochemical studies reported altered serum lysosomal enzyme activity in patients with ALS [77]. However, these peripheral biochemical findings do not directly establish impaired autophagic flux in motor neurons and should be distinguished from the ALS genetic and experimental evidence for altered protein and organelle quality-control pathways discussed above.

Autophagy dysfunction is closely linked to ALS proteinopathy, including TDP-43 pathology. In cellular models, soluble TDP-43 is cleared predominantly by the ubiquitin–proteasome system, whereas clearance of aggregated TDP-43 depends more strongly on autophagy [76]. Genetic studies of *OPTN*, *SQSTM1*/p62, and *TBK1* provide complementary evidence connecting ALS susceptibility to selective autophagy and related protein-clearance pathways [20,23,27]. However, these experimental and genetic associations do not establish that impaired autophagy initiates TDP-43 pathology in all forms of ALS; the direction and timing of the interactions may differ across genotypes and disease stages.

### 5.3. Mitochondrial Dysfunction and Impaired Axonal Homeostasis

Mitochondrial dysfunction is a central feature of ALS and represents a major point of convergence for genetically diverse disease mechanisms. Motor neurons are particularly vulnerable to mitochondrial impairment because of their exceptional metabolic demands and dependence on efficient ATP production, calcium buffering, and long-distance organelle transport. Alterations in mitochondrial dynamics, bioenergetics, oxidative phosphorylation, and quality control have been reported across both familial and sporadic ALS, regardless of the initiating genetic defect [1,11,73,78].

Several ALS-associated genes contribute directly or indirectly to mitochondrial and axonal homeostasis. *CHCHD10* variants can impair mitochondrial cristae organization and respiratory-chain function, whereas *SOD1*, *TARDBP*, *FUS*, *C9ORF72* and *VCP* influence mitochondrial morphology, transport, calcium homeostasis, or mitophagy. Dysfunction of *TBK1*, *OPTN*, and related autophagy genes can further compromise mitochondrial quality control. Genetic evidence also directly implicates the microtubule cytoskeleton: rare *TUBA4A* variants have been associated with ALS, and functional studies demonstrated defective microtubule incorporation and reduced microtubule dynamics and stability, with several variants exerting dominant-negative effects [6,25,27,31,73].

Because mitochondria must be continuously transported along axons to meet local energy requirements, defects in axonal trafficking further amplify neuronal vulnerability. Impaired transport limits ATP availability at distal synapses, disrupts calcium buffering, and compromises axonal maintenance, creating a self-reinforcing cycle of energetic failure and neurodegeneration. Consequently, mitochondrial dysfunction should not be viewed as an isolated pathogenic mechanism but as part of an interconnected network linking RNA metabolism, proteostasis, autophagy, and axonal homeostasis.

### 5.4. Neuroinflammation and Immune Dysregulation

Neuroinflammatory responses are consistently observed in ALS and may contribute to disease progression, although their temporal position and causal contribution are likely to vary across genetic and disease contexts. Activated microglia, reactive astrocytes, infiltrating immune cells, and complement-related pathways can influence neuronal injury and tissue responses [79,80,81,82].

Several ALS-associated genes participate directly in innate immune regulation. *TBK1*, *OPTN*, *CYLD*, and *C9ORF72* modulate autophagy-dependent immune responses, interferon signaling, cytokine production, and microglial activation. Pathogenic variants affecting these pathways can therefore amplify inflammatory responses while simultaneously impairing cellular mechanisms responsible for protein and organelle quality control, further linking immune dysregulation to defective proteostasis and mitochondrial dysfunction.

Increasing evidence also supports bidirectional interactions between neuroinflammation and other pathogenic pathways. Persistent inflammatory activation promotes oxidative stress, protein aggregation, and mitochondrial damage, whereas dysfunctional mitochondria and aggregated proteins act as danger-associated molecular signals that further stimulate innate immune responses. This reciprocal amplification contributes to progressive motor neuron loss and helps explain why inflammation becomes increasingly prominent during disease progression [3,81,82].

Collectively, these findings indicate that neuroinflammation represents an integral component of the ALS pathogenic network rather than an independent downstream process. Therapeutic strategies aimed at modulating inflammatory pathways are therefore likely to be most effective when combined with interventions targeting complementary mechanisms, including RNA dysregulation, impaired proteostasis, and mitochondrial dysfunction.

### 5.5. Systems Biology and Multi-Omics Integration

The increasing complexity of ALS genetics has highlighted the limitations of approaches focused on single genes or isolated molecular pathways. Systems biology provides a framework for integrating genomics, transcriptomics, epigenomics, proteomics, metabolomics, and single-cell technologies to reconstruct molecular networks relevant to motor-neuron degeneration [6,38,83].

Recent advances in single-cell and spatial transcriptomics have revealed cell type-specific molecular alterations that are masked in bulk tissue analyses. Motor neurons, astrocytes, microglia, oligodendrocytes, and other neural cell populations exhibit distinct transcriptional responses during disease progression, supporting the concept that ALS arises from dynamic interactions between neuronal and non-neuronal cells rather than purely cell-autonomous mechanisms [39,84,85].

Proteomic approaches provide an additional layer for linking genetic variation to downstream molecular phenotypes in ALS. Large-scale profiling of plasma and disease-relevant cellular models has identified ALS-associated protein signatures and candidate biomarker panels. Recent studies combining proteomics with machine learning have further illustrated the potential of these data for disease classification, molecular stratification, and identification of altered pathways and candidate biomarkers [86,87]. A targeted plasma proteomics study of *KIF5A*-associated SPG10 and ALS identified phenotype-associated protein signatures involving synaptic and inflammatory pathways, with elevated neurofilament light chain in *KIF5A*-associated ALS but not SPG10 [88].

Machine-learning and deep-learning approaches are increasingly being applied to high-dimensional ALS datasets. Supervised machine learning has been used to derive proteomics-based classifiers, while deep multi-omics approaches have identified molecular subtypes characterized by combinations of cellular and pathological features. Integrative frameworks combining genomic, transcriptomic, proteomic, and clinical information may therefore improve molecular classification and hypothesis generation [89,90]. Multimodal strategies integrating molecular, fluid, imaging, and clinical biomarkers may also contribute to patient stratification, disease monitoring, therapeutic-response assessment, and clinical-trial design [91]. However, these approaches remain predominantly investigational, and independent prospective validation, methodological standardization, reproducibility across platforms and ancestries, and demonstration of clinical utility will be required before routine implementation. A broader methodological review of neuroproteomics has likewise discussed the application of machine learning and deep learning to high-dimensional proteomic datasets across neurological disorders, while highlighting the importance of robust validation [92].

### 5.6. From Convergent Pathways to Therapeutic Targets

Recognition of convergent pathogenic pathways has fundamentally changed therapeutic strategies in ALS. While mutation-specific treatments directly target the initiating genetic defect and provide high biological precision, pathway-directed therapies aim to correct downstream abnormalities shared across genetically diverse forms of ALS, potentially benefiting a much broader patient population.

RNA-based therapies represent a major advance in genetic precision medicine. The accelerated approval of tofersen for *SOD1*-associated ALS demonstrates the translational potential of molecular diagnosis, while other RNA-directed approaches, including *FUS*-targeted antisense therapy, remain under clinical evaluation [93,94,95]. However, clinical development has not been uniformly successful. The *C9ORF72*-targeting antisense oligonucleotide BIIB078 was discontinued after a phase 1 trial showed no reduction in neurofilament levels or clinical benefit relative to placebo [96]. Similarly, development of the *ATXN2*-lowering antisense oligonucleotide BIIB105 (ALSpire, phase 1/2) was discontinued despite reduced cerebrospinal-fluid ataxin-2 concentrations, because neither plasma neurofilament light chain nor clinical measures of function, respiration, and strength improved [97]. These negative findings underscore that target engagement does not necessarily translate into modification of ALS progression and that therapeutic programs must be distinguished by their actual clinical-trial status.

Equally important are pathway-based approaches designed to restore proteostasis, enhance autophagy, improve mitochondrial function, correct RNA-processing abnormalities, regulate neuroinflammation, and preserve axonal integrity. Because these mechanisms are shared across multiple genetic forms of ALS, they may provide broader therapeutic applicability than mutation-specific interventions alone.

The development of reliable biomarkers will be critical for implementing pathway-directed therapies. Although neurofilament light chain has emerged as a robust marker of neuronal injury [98], future multimodal biomarker strategies integrating molecular, fluid, imaging, and clinical measures may improve patient stratification, disease monitoring, therapeutic-response assessment, and clinical-trial design. Their clinical utility, however, requires prospective validation and standardization [86,91].

Future therapeutic strategies will likely combine mutation-specific interventions with treatments targeting convergent pathogenic pathways. This integrated approach has the potential to maximize therapeutic efficacy by simultaneously addressing the initiating genetic defect and the downstream biological processes responsible for disease progression.

### 5.7. Integrated Pathogenic Networks

Although ALS-associated genes were initially assigned to distinct biological functions, it is now evident that these categories overlap extensively. RNA dysregulation promotes protein aggregation, defective proteostasis impairs mitochondrial homeostasis, dysfunctional autophagy amplifies neuroinflammation, and chronic inflammatory responses further compromise neuronal function. Consequently, ALS should be viewed as a disorder of interconnected biological networks rather than a collection of independent pathogenic mechanisms. The principal convergent pathways and representative genes are summarized in Table 3.

Despite increasing evidence for convergence on common cellular pathways, the temporal and causal roles of many of these processes remain incompletely resolved. Specific experimental observations illustrate why a single causal sequence cannot yet be assumed. Experimental depletion of nuclear TDP-43 establishes a loss of splicing repression with cryptic exon inclusion [42,44]. Separately, cell-based protein-clearance experiments indicate that the ubiquitin–proteasome system preferentially removes soluble TDP-43, whereas autophagy contributes more to clearance of aggregated species [76]. These observations are complementary rather than conflicting: they examine different processes, often in experimental systems that do not reproduce the full temporal evolution of human ALS. Although nuclear depletion and cytoplasmic aggregation frequently coexist in affected patient tissue, the cited studies do not establish whether loss of nuclear function precedes aggregation, whether impaired clearance initiates or follows aggregate formation, or whether a single causal sequence applies across ALS genotypes. Direct longitudinal evidence resolving this order in patients remains unavailable. Likewise, ALS-associated *TIA1* variants have been shown experimentally to alter phase separation and stress-granule dynamics [34], but this does not establish that stress granules are obligatory precursors of TDP-43 inclusions in all ALS subtypes. Finally, the genotype-dependent enhancement of *UNC13A* cryptic splicing by ALS risk variants [49,50] contrasts with *STMN2* mis-splicing following TDP-43 depletion [46,47], underscoring that inherited susceptibility and downstream RNA pathology need not coincide. Thus, evidence from different models supports several interacting mechanisms, but their causal order, relative importance, and generalizability across ALS genotypes remain uncertain. These uncertainties caution against a strictly linear model of ALS pathogenesis and instead support dynamic interactions among partially overlapping processes whose relative contributions differ across biological contexts.

This system-level perspective explains why genetically diverse patients frequently develop remarkably similar pathological and clinical phenotypes despite carrying different primary mutations. It also provides a biological framework for understanding incomplete penetrance, variable expressivity, and the cumulative effects of multiple genetic and environmental factors, all of which influence the resilience of motor neurons and surrounding glial cells.

Importantly, network-based interpretation also improves variant assessment by placing rare or incompletely characterized variants within their biological context. Variants affecting proteins that participate in repeatedly implicated molecular pathways may warrant greater consideration than isolated findings, although pathway membership alone cannot establish pathogenicity and must always be integrated with population, segregation, and functional evidence.

Ultimately, the convergence of heterogeneous genetic defects on shared molecular networks provides the conceptual bridge between monogenic and genetically complex ALS. Integrating genomic, transcriptomic, proteomic, metabolomic, and functional data will be essential to define biologically meaningful patient subgroups and develop precision medicine strategies that combine mutation-specific therapies with interventions targeting shared pathogenic vulnerabilities.

## 6. Clinical Implications and Future Perspectives

The expanding understanding of ALS genetic architecture is reshaping molecular diagnosis, genetic counseling, biomarker development, and therapeutic strategies. Beyond conventional gene panels, whole-genome sequencing (WGS), long-read sequencing, transcriptomics, and functional genomic approaches are increasingly identifying pathogenic mechanisms that escape standard diagnostic testing, improving diagnostic yield and refining interpretation of variants of uncertain significance [6,37,38,39].

These advances also transform genetic counseling. Recognition of non-coding variants, structural variants, repeat expansions, oligogenic inheritance, and genetic modifiers demonstrates that disease risk often reflects the combined effects of multiple genetic and non-genetic factors rather than a single pathogenic mutation. Consequently, future counseling will require integrated interpretation of genomic findings together with family history, clinical phenotype, ancestry, age-dependent penetrance, and functional evidence [99,100].

As genomic testing expands, variant interpretation has become the principal diagnostic challenge. Many regulatory, deep-intronic, structural, and repeat variants cannot be classified confidently using DNA sequence alone and require transcriptomic analyses, segregation studies, functional assays, and standardized interpretation frameworks for clinical implementation [101].

These diagnostic advances parallel the emergence of precision therapeutics. The accelerated approval of tofersen for *SOD1*-associated ALS established proof of principle for genetically targeted intervention [95,102]. Nevertheless, the clinical pipeline must be interpreted considering both ongoing investigations, including *FUS*-directed antisense approaches, and discontinued programs. BIIB078 for *C9ORF72*-associated ALS and BIIB105 targeting *ATXN2* were discontinued after trials failed to demonstrate meaningful clinical benefit despite evidence of molecular target engagement in the latter program [96,97]. These outcomes highlight the need for disease-relevant pharmacodynamic biomarkers and rigorous clinical efficacy assessment, while therapies directed against shared pathogenic pathways may eventually extend treatment opportunities beyond mutation-specific subgroups.

Reliable biomarkers will be essential for implementing precision medicine. Beyond neurofilament light chain, future biomarker panels integrating genomic, transcriptomic, proteomic, metabolomic, imaging, and digital phenotyping data are expected to improve patient stratification, monitor target engagement, predict therapeutic response, and support biomarker-guided clinical trials.

Despite these advances, important challenges remain, including unresolved molecular diagnoses, limited functional validation of rare variants, underrepresentation of non-European populations, and the integration of multidimensional molecular datasets into clinically actionable models. Addressing these limitations will require international collaboration, standardized variant interpretation, and ancestry-diverse genomic studies.

Ultimately, the transition from a monogenic to a multilayer model of ALS genetics is redefining clinical practice. Integrating comprehensive molecular profiling with functional validation and biomarker-guided patient stratification will provide the foundation for next-generation precision medicine, in which mutation-specific therapies and interventions targeting shared pathogenic pathways are combined to optimize individualized treatment.

## 7. Outstanding Challenges and Research Priorities

Despite remarkable advances in ALS genetics, several key challenges remain. Future research should focus not only on identifying additional disease-associated variants but also on understanding how different classes of genomic variation interact to influence disease susceptibility, phenotypic heterogeneity, and progression [6,7,38].

A major priority is the comprehensive characterization of genetic variation that remains poorly detected by conventional sequencing approaches. Deep-intronic variants, structural rearrangements, repeat expansions, mobile-element insertions, and variants within repetitive genomic regions are still incompletely captured by current diagnostic pipelines. Wider implementation of whole-genome sequencing, long-read sequencing, and complementary genomic technologies will improve variant detection, but accurate interpretation will continue to require functional validation and integrated multi-omics analyses [37,38,39].

Another important challenge is defining the contribution of somatic mosaicism and tissue-specific genomic variation. Post-zygotic mutations, repeat instability, and epigenetic differences between blood and nervous tissue may contribute to selective neuronal vulnerability but remain poorly characterized. High-depth sequencing, long-read technologies, single-cell approaches, and analyses of disease-relevant tissues will be essential to address this question [57,58,64].

Functional interpretation of non-coding and regulatory variants also remains a major limitation. Integrating DNA sequencing with transcriptomics, epigenomics, chromatin profiling, proteomics, and disease-relevant cellular models will be critical for distinguishing pathogenic variants from benign genomic variation. The TDP-43-dependent regulation of *STMN2* and *UNC13A* exemplifies how functional analyses can reveal pathogenic mechanisms that cannot be inferred from DNA sequence alone [44,46,47,49,50].

Equally important is the inclusion of ancestrally diverse populations. The predominance of European cohorts limits variant interpretation, reduces the transferability of polygenic risk models, and contributes to disparities in molecular diagnosis. Expanding global genomic initiatives will improve variant classification and reveal population-specific mechanisms of disease [55,71].

Finally, future progress will depend on integrating genomic, transcriptomic, epigenomic, proteomic, metabolomic, functional, environmental, and longitudinal clinical data into unified analytical frameworks. Machine-learning and network-based approaches may facilitate variant prioritization, molecular stratification, and biomarker discovery, but their clinical implementation will require prospective validation, methodological standardization, diverse cohorts, reproducibility, and integration with experimental and clinical evidence.

Ultimately, the next phase of ALS research will depend less on expanding gene catalogues than on understanding how diverse genetic mechanisms converge within interconnected biological networks. This system-level perspective will be essential for improving molecular diagnosis, refining disease classification, identifying new therapeutic targets, and advancing precision medicine.

## 8. Conclusions

The understanding of ALS genetics has evolved from a predominantly monogenic framework to a multilayer model of disease architecture. Although the discovery of highly penetrant pathogenic variants in *SOD1*, *TARDBP*, *FUS*, and *C9ORF72* has transformed our understanding of motor neuron degeneration and enabled the first genetically targeted therapies, monogenic inheritance explains only part of ALS susceptibility and cannot fully account for incomplete penetrance, apparently sporadic disease, or the marked clinical heterogeneity observed among patients [1,3,4,6,11,12].

Current evidence indicates that ALS susceptibility arises from the combined contribution of multiple forms of genomic variation, including non-coding variants, structural rearrangements, repeat expansions, oligogenic interactions, common susceptibility alleles, and genetic modifiers. Together with ageing, ancestry, and environmental influences, these factors shape a continuum extending from highly penetrant Mendelian disease to genetically complex forms characterized by cumulative genetic liability [6,7,12,68,103].

Despite this genetic diversity, ALS-associated alterations consistently converge on a limited number of interconnected biological processes, including RNA metabolism, proteostasis, autophagy, mitochondrial homeostasis, axonal transport, and neuroinflammatory signaling. This convergence explains how distinct genetic defects produce similar pathological and clinical phenotypes while identifying shared molecular vulnerabilities that may be therapeutically targeted across genetically heterogeneous patient populations [1,3,11,73].

Recent advances in whole-genome sequencing, long-read sequencing, transcriptomics, and functional genomics are expanding the spectrum of detectable pathogenic variation. However, future progress will depend less on identifying additional disease genes than on integrating genomic discoveries with transcriptomic, functional, and longitudinal clinical evidence to improve variant interpretation, refine patient stratification, and define biologically meaningful therapeutic targets [38,39,83].

Ultimately, ALS should be viewed as a spectrum of disorders in which diverse forms of genomic variation interact with genetic background and other modifiers and converge, to varying degrees, on interconnected biological networks. Moving beyond a purely monogenic framework toward an integrated, multilayer model of ALS genetics may improve molecular diagnosis and patient stratification and inform mechanism-based therapeutic development. Translation of these approaches into routine precision medicine will nevertheless require prospective validation, standardization, reproducibility, and demonstration of clinically meaningful utility.

## Figures and Tables

**Table 1:** Major ALS-associated genes, principal biological functions, and representative pathogenic mechanisms.

Gene	Main Biological Function	Representative Pathogenic Mechanism	Genetic Evidence	Representative References
*C9ORF72*	Autophagy, endolysosomal trafficking, immune regulation	Repeat expansion, RNA toxicity, DPR proteins, haploinsufficiency	Definitive	[9,10]
*SOD1*	Oxidative stress defense	Toxic protein misfolding and aggregation	Definitive	[8]
*TARDBP*	RNA metabolism, splicing	Nuclear depletion, cytoplasmic aggregation, cryptic exon inclusion	Definitive	[14]
*FUS*	RNA processing, DNA repair	RNA dysregulation, protein aggregation	Definitive	[16]
*TBK1*	Selective autophagy, innate immunity	Impaired autophagy and neuroinflammation	Definitive	[27]
*OPTN*	Autophagy, mitophagy	Defective protein/organelle clearance	Definitive	[20]
*SQSTM1* (p62)	Ubiquitin-mediated autophagy	Impaired proteostasis	Moderate	[23]
*VCP*	Protein degradation, ER-associated degradation	Defective protein quality control	Definitive	[19]
*UBQLN2*	Ubiquitin–proteasome pathway	Protein aggregation	Definitive	[22]
*NEK1*	DNA repair, cytoskeleton	Impaired DNA damage response and axonal maintenance	Definitive	[28]
*KIF5A*	Axonal transport	Defective intracellular transport	Definitive	[29]
*SPG11*	Lysosomal function, axonal maintenance	Impaired lysosomal trafficking and axonal degeneration	Tenuous	[21]
*CHCHD10*	Mitochondrial homeostasis	Mitochondrial dysfunction	Definitive	[31]
*ANXA11*	RNA granules, vesicle trafficking	Altered phase separation and RNA granule dynamics	Definitive	[32]
*MATR3*	RNA metabolism	Altered RNA processing	Tenuous	[24]
*HNRNPA1/HNRNPA2B1*	RNA metabolism, stress granules	Aberrant RNA processing and phase separation	Tenuous/Tenuous	[33]
*TIA1*	Stress-granule dynamics	Persistent stress granules and protein aggregation	Tenuous	[34]
*DNAJC7*	Protein folding (HSP40 co-chaperone)	Impaired protein quality control	Moderate	[30]
*TUBA4A*	Microtubule organization and cytoskeletal stability	Impaired microtubule assembly, dynamics, and stability	Strong	[25]

DPR = dipeptide repeat proteins; ER = endoplasmic reticulum.

**Table 2:** Principal classes of hidden genomic variation in ALS, detection strategies, and clinical implications.

Class of Genomic Variation	Typical Examples	Main Detection Methods	Potential Clinical Implications	Representative References
Non-coding variants	Promoters, enhancers, UTRs, deep-intronic variants	WGS, RNA sequencing, splicing assays	Aberrant gene regulation and cryptic exon inclusion	[49,50]
Structural variants	CNVs, insertions, deletions, inversions, translocations	WGS, long-read sequencing, optical genome mapping	Gene disruption and altered chromatin architecture	[54,55]
Repeat expansions	*C9ORF72*, *ATXN2*, *NIPA1*	Repeat-primed PCR, Southern blot, long-read sequencing	RNA toxicity, DPR proteins, somatic instability	[9,10,59]
Regulatory variants	TF-binding and chromatin regulatory variants	WGS, epigenomic profiling	Altered transcriptional regulation	[12]
Splicing variants	Cryptic splice-site and intronic variants	RNA sequencing, transcript analysis	Aberrant RNA processing	[49,50]
Complex rearrangements	Complex SVs and chromosomal events	Long-read sequencing, optical genome mapping	Previously undetected pathogenic mechanisms	[38,39]

CNV = copy-number variant; DPR = dipeptide repeat proteins; PCR = polymerase chain reaction; SV = structural variant; UTR = untranslated region; WGS = whole-genome sequencing.

**Table 3:** Convergent pathogenic pathways and representative ALS-associated genes.

Pathogenic Pathway	Representative ALS-Associated Genes	Major Cellular Consequences	Representative References
RNA metabolism and RNA processing	*TARDBP*, *FUS*, *MATR3*, *HNRNPA1*, *HNRNPA2B1*, *TIA1*	Aberrant splicing, cryptic exon inclusion, impaired RNA transport, stress-granule dysfunction	[42,43]
Proteostasis and protein degradation	*SQSTM1*, *OPTN*, *VCP*, *UBQLN2*, *TBK1*	Protein aggregation, impaired ubiquitin–proteasome system, defective autophagy	[20,22,23,27,76]
Autophagy–lysosome pathway	*TBK1*, *OPTN*, *SQSTM1*, *C9ORF72*, *SPG11*	Impaired autophagic flux, defective lysosomal degradation, abnormal organelle clearance	[20,23,27,76]
Mitochondrial homeostasis	*CHCHD10*, *SOD1*, *FUS*, *TARDBP*, *VCP*	Mitochondrial dysfunction, oxidative stress, impaired energy metabolism	[31,73]
Axonal transport and neuronal maintenance	*KIF5A*, *SPG11*, *NEK1*, *TUBA4A*	Defective axonal trafficking, impaired neuronal maintenance, distal axon degeneration	[25,29]
Neuroinflammation and innate immunity	*TBK1*, *OPTN*, *C9ORF72*, *CYLD*	Microglial activation, dysregulated innate immunity, chronic neuroinflammation	[81,82]
DNA damage response	*NEK1*, *FUS*, *TARDBP*	Impaired DNA repair, genomic instability	[16,28]
Integrated pathogenic network	Multiple ALS genes	Functional convergence of RNA dysregulation, proteostasis failure, mitochondrial dysfunction, autophagy defects and neuroinflammation leading to progressive motor neuron degeneration	[3,11]

## Data Availability

No new data were created or analyzed in this study. Data sharing is not applicable to this article.
